# Selection, Characterization and Interaction Studies of a DNA Aptamer for the Detection of *Bifidobacterium bifidum*

**DOI:** 10.3390/ijms18050883

**Published:** 2017-04-25

**Authors:** Lujun Hu, Linlin Wang, Wenwei Lu, Jianxin Zhao, Hao Zhang, Wei Chen

**Affiliations:** 1 State Key Laboratory of Food Science and Technology, School of Food Science and Technology, Jiangnan University, Wuxi 214122, China; 7130112038@vip.jiangnan.edu.cn (L.H.); wanglllynn09@163.com (L.W.); luwenwei@jiangnan.edu.cn (W.L.); jxzhao@jiangnan.edu.cn (J.Z.); zhanghao@jiangnan.edu.cn (H.Z.); 2 International Joint Research Center for Probiotics & Gut Health, Jiangnan University, Wuxi 214122, China; 3 Beijing Innovation Centre of Food Nutrition and Human Health, Beijing Technology and Business University, Beijing 100048, China

**Keywords:** *Bifidobacterium bifidum*, aptamer, SELEX, sequence truncation, colorimetric bioassay

## Abstract

A whole-bacterium-based SELEX (Systematic Evolution of Ligands by Exponential Enrichment) procedure was adopted in this study for the selection of an ssDNA aptamer that binds to *Bifidobacterium bifidum*. After 12 rounds of selection targeted against *B. bifidum*, 30 sequences were obtained and divided into seven families according to primary sequence homology and similarity of secondary structure. Four FAM (fluorescein amidite) labeled aptamer sequences from different families were selected for further characterization by flow cytometric analysis. The results reveal that the aptamer sequence CCFM641-5 demonstrated high-affinity and specificity for *B. bifidum* compared with the other sequences tested, and the estimated *K*_d_ value was 10.69 ± 0.89 nM. Additionally, sequence truncation experiments of the aptamer CCFM641-5 led to the conclusion that the 5′-primer and 3′-primer binding sites were essential for aptamer-target binding. In addition, the possible component of the target *B. bifidum*, bound by the aptamer CCFM641-5, was identified as a membrane protein by treatment with proteinase. Furthermore, to prove the potential application of the aptamer CCFM641-5, a colorimetric bioassay of the sandwich-type structure was used to detect *B. bifidum*. The assay had a linear range of 10^4^ to 10^7^ cfu/mL (*R*^2^ = 0.9834). Therefore, the colorimetric bioassay appears to be a promising method for the detection of *B. bifidum* based on the aptamer CCFM641-5.

## 1. Introduction

Aptamers are highly structured single-stranded oligonucleotides obtained from an in vitro evolution process called Systematic Evolution of Ligands by Exponential Enrichment (SELEX) according to their binding abilities to target molecules [1,2]. Compared with traditional antibodies, aptamers have many advantages such as low molecular weight, ease of synthesis and modifications, and comparable stability during long-term storage [3,4,5]. Whole-bacterium SELEX was specifically developed to separate aptamers against live bacteria, and it is a particularly promising selection strategy for the identification of bacteria. Bacterium-based aptamer selection methods have been implemented to select ssDNA aptamers against many bacteria, including *Campylobacter jejuni*, *Escherichia coli*, *Lactobacillus acidophilus*, *Mycobacterium tuberculosis*, *Vibrio parahemolyticus*, *Streptococcus pyogenes*, and *Staphylococcus aureus* without previous knowledge of a specific target molecule [6,7,8,9,10,11,12].

Many studies have found that *Bifidobacterium bifidum* has the potential to prevent inflammatory bowel disease and necrotizing enterocolitis, reduce cholesterol activity, treat infantile eczema, modulate the host innate immune response, show preventive potential for diarrheal disease in infants, and exert a key role in the evolution and maturation of the immune system of the host [13,14,15,16,17,18,19]. *B. bifidum* has also been granted QPS (quality and presumption of safety) status by the European Food Safety Authority. Thus, *B. bifidum* is often used in probiotic products along with other lactic acid bacteria [20,21,22], and identification of *B. bifidum* is vital for its industrial use. The conventional approaches for the identification of *B. bifidum* are laborious and time-consuming. There is thus a need of developing alternative methods for the identification of *B. bifidum*. Many molecular methods have been developed for the identification of *B. bifidum* [23,24,25,26], but they increase the analysis cost in that they require specialized instruments and highly trained personnel [24,27,28]. Therefore, aptamers may be an alternative method for the detection of *B. bifidum*.

Enzyme linked aptamer assay (ELAA), a variant of the classical ELISA (enzyme linked immunosorbent assay) uses aptamers instead of antibodies [29,30], and uses an enzyme as the signal readout element and an aptamer as the recognition element. ELAA can realize high-throughput with a 96-well microplate and is convenient because the signal readout requires only simple instruments (or even no instruments, when read with the naked eye). Therefore, ELAA has been used in many bioanalytical applications for target-specific detection of some substances such as *M. tuberculosis*, ochratoxin A, cocaine and thrombin [31,32,33,34]. However, ELAA has not been reported in the detection of *B. bifidum*.

In this study, we used an improved whole-bacterium SELEX strategy to select an ssDNA aptamer specific for *B. bifidum*. In addition, truncation experiments were carried out to narrow down the sequence region of the potential aptamers essentially for their binding abilities to the target *B. bifidum*. In addition, *B. bifidum* was treated with proteinases to determine whether the targets of the aptamer were the membrane proteins on the cell surface of *B. bifidum*. Furthermore, to confirm the potential application of the candidate aptamer, we developed a colorimetric assay that was a high-throughput, sensitive and specific method for the detection of *B. bifidum*.

## 2. Results and Discussion

### 2.1. SELEX Optimization

To separate aptamers that specifically recognize *B. bifidum*, we gradually increased the selective pressure by increasing bovine serum albumin (BSA) and tRNA from a 10-fold molar excess of each in the starting round of selection to a maximum 120-fold molar excess in the 12th round, and by increasing the number of washes (from twice for the first six cycles to three times for the last six cycles). In addition, the suspended cell solutions were transferred to fresh microcentrifuge tubes to remove sequences that bound to the tube walls between each incubation and elution step. Counter-selection against a mixture of unrelated *Bifidobacterium* species, including *B. longum*, *B. animalis*, *B. breve*, and *B. adolescentis*, was employed in the 9th and 11th rounds. In addition, 2.5 μL DMSO (dimethyl sulfoxide) was chosen for the total volume of 50 μL during the PCR amplification of SELEX.

### 2.2. Determination of Affinity and Specificity

Fluorescently labeled aptamer sequences were incubated with *B. bifidum* and tested via flow cytometric analysis. After the 12th round of selection, 30 sequences were obtained after the aptamer pools were cloned and sequenced. These sequences were then grouped into seven families according to the homology of the DNA sequences and the similarity of the secondary structure (data shown in Table S1 and Figure S1 in the Supplemental Materials). Four sequences were selected for further screening on the basis of their repetitiveness, predicted secondary structure and free energy of formation (Table 1).

**Figure 1:**
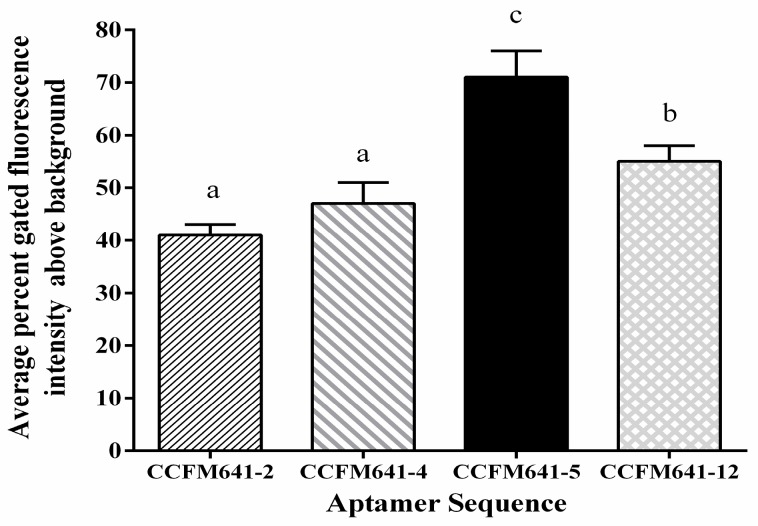
Binding affinity of aptamers for *B. bifidum*. The 5′-FAM-labeled individual aptamers were incubated with *B. bifidum* at 37 °C for 45 min (as described in the text). The values of aptamer binding represent the mean ± SD of three independent experiments. Bars with different letters are significantly different (*p* < 0.05).

**Figure 2:**
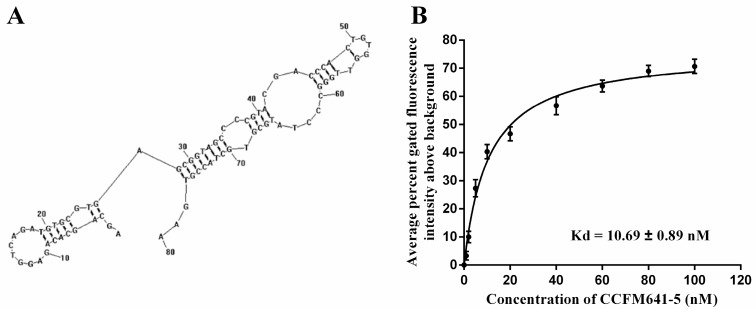
The secondary structure and binding ability of aptamer CCFM641-5 against *B. bifidum*. (**A**) The secondary structure of aptamer CCFM641-5. The secondary structure was predicted using RNAstructure 3.0. (**B**) The binding saturation curve of aptamer CCFM641-5 with *B. bifidum*. A nonlinear regression curve was fit according to the data from flow cytometric analysis using GraphPad Prism 5.0. The values of aptamer binding and *K*_d_ represent the mean ± SD of three independent experiments.

The results displayed in Figure 1 demonstrated that CCFM641-5 showed a stronger binding affinity for *B. bifidum* than the other three aptamers. To further evaluate the binding ability of the aptamer CCFM641-5 to the target *B. bifidum*, we performed binding assays by varying the concentrations of the aptamer (from 0 to 100 nM) and using a constant number of cells (10^8^ cells) for each assay. Saturation curves were fit from these data and the dissociation constant *K*_d_ values were determined via nonlinear regression analysis. The dissociation constant *K*_d_ between CCFM641-5 and *B. bifidum* was calculated to be 10.69 ± 0.89 nM. Therefore, aptamer CCFM641-5 was chosen for the specificity detection. The predicted secondary structure and binding saturation curve of aptamer CCFM641-5 for *B. bifidum* are shown in Figure 2.

**Figure 3:**
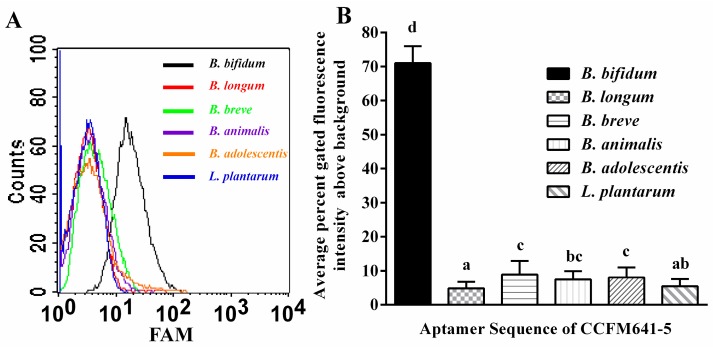
Characterization of the specificity of aptamer CCFM641-5 for *B. bifidum*. Selected aptamer sequence CCFM641-5 preferentially bound to *B. bifidum* over other species of bacteria. (**A**) Flow cytometric analysis of aptamer CCFM641-5 binding for different species of bacteria which are shown with differently colored curves; (**B**) Histogram of the percent gated fluorescence intensity above background for aptamer CCFM641-5. The values of aptamer binding represent the mean ± SD of three independent experiments. Bars with different letters are significantly different (*p* < 0.05).

To determine the specificity of the candidate aptamer CCFM641-5 for the target *B. bifidum*, the FAM (fluorescein amidite) labeled aptamer CCFM641-5 was also tested against a variety of other bacterial species, including *B. longum*, *B. animalis*, *B. breve*, *B. adolescentis* and *L. plantarum*. As shown in Figure 3, the aptamer CCFM641-5 displayed preferential binding ability to *B. bifidum* over the other bacteria tested. In addition, the results from qPCR shown in Figure S2 demonstrated that CCFM641-5 displayed a stronger binding ability to *B. bifidum* than the other bacterial species. Taken together, this preferential binding demonstrated the excellent specificity of the aptamer CCFM641-5 for *B. bifidum*.

### 2.3. Aptamer Truncations and Their Effects on the Binding Ability to B. bifidum

**Figure 4:**
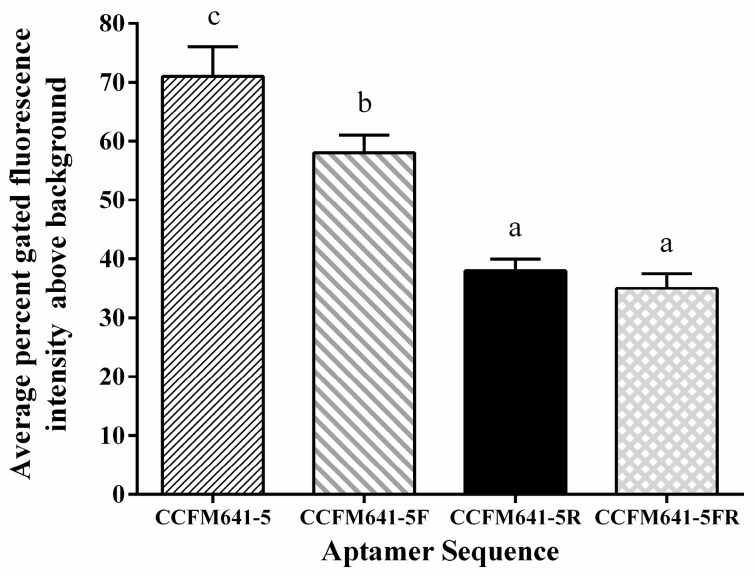
Binding abilities of the truncated aptamer variants to *B. bifidum* compared to the full-length aptamer CCFM641-5. The values of aptamer binding represent the mean ± SD of three independent experiments. Bars with different letters are significantly different (*p* < 0.05).

In general, not all nucleic acids of the aptamers are necessary for binding affinity between the aptamers and the targets [35]. To determine the minimal sequence necessary for binding affinity between *B. bifidum* and the aptamer CCFM641-5, the aptamer was truncated to narrow down the sequence region responsible for target binding affinity. Either specific primer binding site at the ends of aptamer CCFM641-5 (CCFM641-5F and CCFM641-5R) or both sites (CCFM641-5FR) were removed (Table 2). As displayed in Figure 4, all the aptamer variants bound to *B. bifidum* with a lower binding affinity compared to the full-length aptamer CCFM641-5. Taken together, the results indicate that the 3′-primer and 5′-primer binding sites of aptamer CCFM641-5 are important for its binding affinity for *B. bifidum*, even if the aptamer affinity is still largely preserved for the 5′ truncation.

### 2.4. Proteinase Treatment for Bacteria

**Figure 5:**
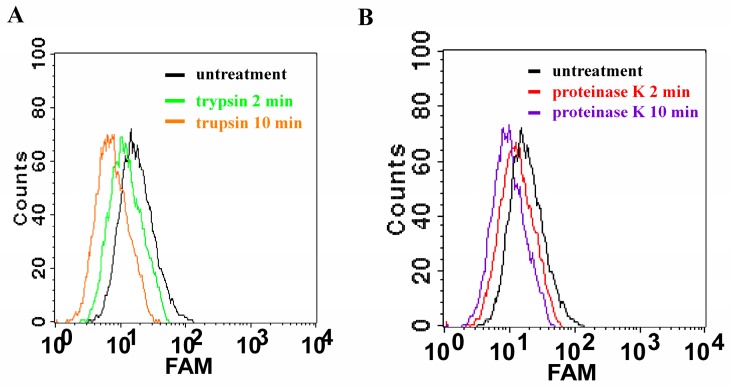
Binding assays of aptamer CCFM641-5 to trypsin-treated or proteinase K-treated *B. bifidum*. (**A**) Flow cytometric analysis of aptamer CCFM641-5 binding affinity for trypsin-treated *B. bifidum*; (**B**) Flow cytometric analysis of aptamer CCFM641-5 binding ability to proteinase K-treated *B. bifidum*.

To evaluate that the targets of the aptamer are membrane proteins on the *B. bifidum* cell surface, we treated *B. bifidum* with proteinases including trypsin and proteinase K for a short time before adding the aptamer CCFM641-5 to these treated bacteria. As revealed in Figure 5, after the bacteria were treated with trypsin or proteinase K for 2 and 10 min, respectively, in phosphate-buffere saline (PBS, pH 7.2) at 37 °C, aptamer CCFM641-5 lowered its binding ability to *B. bifidum*. It can be deduced that the binding entities of aptamer CCFM641-5 had been broken by the proteinases, suggesting that the target molecules are in fact membrane proteins.

### 2.5. Colorimetric Detection of B. bifidum

**Figure 6:**
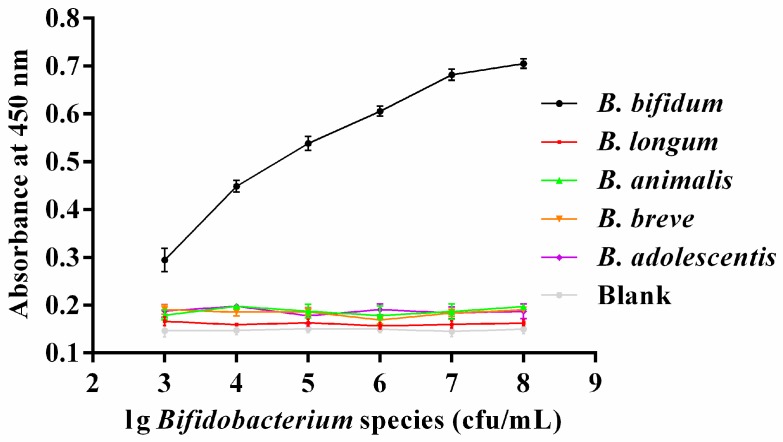
The absorbance at 450 nm measured for different species of bifidobacteria at concentrations ranging from 10^3^ to 10^8^ cfu/mL. The absorbance at 450 nm represents the mean ± SD of three independent experiments.

**Figure 7:**
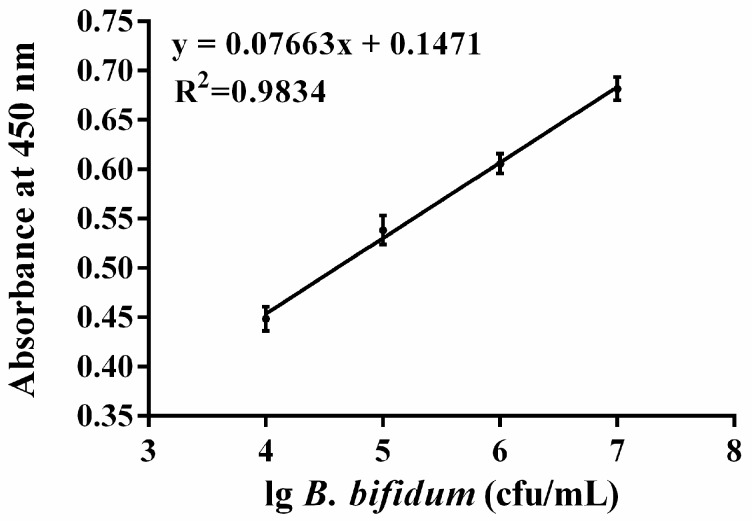
The calibration curve between the concentrations of *B. bifidum* and the intensity of the signals. The OD values were determined by the microplate reader at 450 nm wavelength. The absorbance at 450 nm represents the mean ± SD of three independent experiments.

In this study, the candidate aptamer CCFM641-5 was used not only to capture but also to detect *B. bifidum* in the configuration of the colorimetric assay. In the assay, a sandwich-type structure of aptamer/target/aptamer was established. To evaluate the specificity of this method, one blank sample and five samples including *B. bifidum*, *B. longum*, *B. animalis*, *B. breve* and *B. adolescentis* were measured. The assays of all samples were carried out under the same conditions, and the concentrations of all bacteria were between 10^3^ and 10^8^ cfu/mL. As shown in Figure 6, the optical density (OD) values at 450 nm of the blank sample and the four bacteria other than *B. bifidum* did not change when the concentrations of bacteria were increased, whereas the OD values at 450 nm of *B. bifidum* increased as concentrations of the bacteria increased. Particularly, the results shown in Figure 7 displayed a good linear relationship between the amounts of *B. bifidum* ranging from 10^4^ to 10^7^ cfu/mL and the OD values at 450 nm, with a regression coefficient of 0.9834. The limit of detection of the proposed method was estimated to be 10^4^ cfu/mL at a signal to noise ratio of 3.

## 3. Materials and Methods

### 3.1. Reagents and Apparatus

*B. bifidum* ATCC 29521 was adopted as the target for whole-bacterium SELEX. Other *Bifidobacterium* species used in the study included: *B. longum* ATCC 15697, *B. breve* ATCC 15700, *B. animalis* JCM 11658, and *B. adolescentis* ATCC 15705, which were supplied by the American Type Culture Collection (ATCC) or the Japan Collection of Microorganisms (JCM). All of the *Bifidobacterium* species were grown in de Man–Rogosa–Sharpe (MRS) broth with 0.05% of l-cysteine hydrochloride monohydrate at 37 °C. *L. plantarum* ST-III (CGMCC No. 0847) used in this study was cultured in MRS broth at 37 °C (Merck KGaA, Darmstadt, Germany). All bacterial strains were cultured to the logarithmic phase under anaerobic conditions. 

The starting ssDNA library and the PCR primers used for PCR amplifications were supplied by Integrated DNA Technologies (IDT, Coralville, IA, USA); tRNA, DMSO and streptavidin-HRP (horseradish peroxidase) were purchased from Sigma (St. Louis, MO, USA); BSA and all PCR chemicals were ordered from Invitrogen China (Shanghai, China); trypsin and proteinase K were purchased from TaKaRa (TaKaRa, Dalian, China); and DNA-BIND 96-well plates were obtained from Corning (Corning, New York, NY, USA). Water was filtered with a Milli-Q water purification system (Millipore, Bedford, MA, USA). All reagents were of analytical grade. Purification treatments were carried out with an Eppendorf 5424R centrifuge. PCR amplification was carried out in a Bio-Rad T 100 Thermal Cycler (Bio-Rad Laboratories, Hercules, CA, USA).

### 3.2. DNA Library and PCR Amplification

The 80-nt ssDNA library consisted of a central random region of 40 nucleotides, where equimolar amounts of A, G, C and T are available at each position, flanked by two constant primer binding sequences, and the ssDNA library was synthesized with the following sequence: 5′-AGCAGCACAGAGGTCAGATG-N40-CCTATGCGTGCTACCGTGAA-3′. The ssDNA library and following aptamer pools were amplified with sense (5′-AGCAGCACAGAGGTCAGATG-3′) and antisense primer (5′-TTCACGGTAGCACGCATAGG-3′). 

The PCR amplification mixture was as follows: 1× PCR amplification buffer, 10 μM forward and reverse primer, 25 mM dNTPs, 5 U/μL of Taq DNA polymerase, 2 μL of the template and 2.5 μL of DMSO in a total volume of 50 μL. PCR amplification was initiated with pre-denaturation at 95 °C for 6 min, followed by 25 cycles with 30 s denaturation at 95 °C, 30 s hybridization at 69 °C, 20 s extension at 72 °C, and finally elongation for 5 min at 72 °C. 

PCR amplification products were detected by 8% nondenaturing polyacrylamide gel electrophoresis (PAGE) in 1× TBE buffer (90 mM Tris/89 mM boric acid/2.0 mM EDTA, pH 8.0) (Bio-Rad Protean III, Hercules, CA, USA) at 200 V for 25 min. The polyacrylamide gels were then stained with ethidium bromide, destained with gel running buffer solution, and photographed under UV light. A Qiagen MinElute PCR Purification Kit was used to purify all PCR amplification products (Qiagen Inc., Valencia, CA, USA).

### 3.3. Aptamer Selection

The SELEX procedure for aptamer selection was implemented using the method described previously with some modifications [8,36]. *B. bifidum* was grown in liquid culture media and collected when it reached the logarithmic phase. The cell mixtures were centrifuged at 5000× *g* and 4 °C for 5 min and washed twice with 500 μL PBS at room temperature. The original ssDNA library/pool was heat denatured at 95 °C for 10 min and rapidly chilled for 10 min in an ice bath before incubation. *B. bifidum* cells totaling 10^8^ cfu/mL were incubated with 2 nmol of the ssDNA library for the initial round or 100 pmol of the aptamer pool for subsequent rounds (600 µL for the initial round, 350 µL for subsequent rounds). An excess of tRNA and BSA were put into the incubation buffer. All incubations were implemented in PBS at 37 °C for 45 min with slight agitation. The cells were centrifuged as described above before washing three times in PBS with 0.05% BSA. The cells were then resuspended with 100 μL of 1× PCR reaction buffer, heat denatured at 95 °C for 10 min, snap cooled for 10 min in an ice bath, and extracted by centrifugation as described above, and the supernatant was used as the template for PCR amplification to acquire the ssDNA pool for the following round of selection. 

The ssDNA pool from the 12th round of selection was amplified and then cloned using the TOPO TA Cloning Kit (Invitrogen, Shanghai, China). Individual colonies were picked randomly and their inserts were sequenced. DNAMAN software was adopted to analyze the aptamer sequences and RNAstructure 3.0 was used for predicting a secondary structure for each sequence [10,12,37].

### 3.4. Flow-Cytometric Analysis

A FACSCalibur flow cytometer with a PowerMacG4 workstation and CellQuest Pro software (BD Biosciences, San Jose, CA, USA) was employed to assess the binding ability of the individual aptamer sequences to different species of bacteria (*B. bifidum*, *B. breve* , *B. longum*, *B. animalis*, *B. adolescentis*, and *L. plantarum*) in separate experiments. The aptamers were labeled with FAM fluorophore at the 5′ end. In the binding assays, 10^8^ cells were incubated with the fluorescently labeled aptamer pool (100 nM) at 37 °C for 45 min with slight agitation. The cells were then washed in PBS, collected by centrifugation, and resuspended in PBS for prompt flow cytometric assays. Forward scatter, side scatter, and fluorescence intensity were measured, and the gated fluorescence intensity above the background (cells with no aptamers) was quantified. BD CellQuest Pro software was adopted to analyze data from the FACSCalibur and to create histogram overlays. Binding dissociation constant *K_d_* values were obtained from the binding curves created with GraphPad Prism 5.0 software by varying the aptamer concentration (0 to 100 nM) with a fixed number of cells (10^8^ cells).

### 3.5. Aptamer CCFM641-5 Binding Assays by Quantitative PCR (qPCR)

To further determine binding affinities of aptamer CCFM641-5 for different bacterial species, qPCR was performed as previously described with some modifications [7]. In the binding assays, 10^7^ bacterial cells were incubated with the aptamer (50 nM) at 37 °C for 45 min with slight agitation. The cells were then washed in PBS, collected by centrifugation, and resuspended with 50 μL of 1× PCR reaction buffer. The ssDNA aptamers recovered in the supernatant were used as the template for quantification by SYBR Green-based qPCR using a CFX96 real-time PCR detection system (Bio-Rad Laboratories, Hercules, CA, USA). All qPCR amplifications were carried out in 20 μL volume using 96-well plates in triplicate.

### 3.6. Aptamer Truncation

Truncation experiments were performed to determine whether all nucleotides of the aptamer sequence CCFM641-5 are necessary [38,39]. The specific primer binding sites of the aptamers were first removed. If DNA aptamer variants possessed high binding affinity for *B. bifidum*, the aptamer variants were then truncated from the 5′ end or 3′ end. In the experiment, the truncated aptamer variants were FAM-labeled at the 5′ end and tested for their binding abilities to *B. bifidum* with flow cytometric assays as described above.

### 3.7. Proteinase Treatment for Bacteria

The procedure used in this study was based on a previously published method [9,40] with some modifications, listed as follows. *B. bifidum* (10^8^ cells) was collected by centrifugation, washed twice with PBS and incubated with 1 mL of 0.25% trypsin or 0.1 mg/mL proteinase K in PBS at 37 °C for 2 and 10 min. After incubation, the mixture was washed with PBS, and the treated bacteria were incubated with FAM-labeled aptamer for further binding assays as described above in flow-cytometric analysis.

### 3.8. Colorimetric Bioassay on the Basis of the Selected Aptamer

A colorimetric sandwich-type assay for the detection of *B. bifidum* was developed by ELAA. First, the amino-modified candidate aptamer was dissolved in binding buffer (0.01 mol/L PBS) and 100 μL of diluted aptamer (40 pmol/well) was added into each well of the DNA-BIND 96-well plate for incubation at 37 °C for 1 h. The wells were washed three times with washing buffer (0.01 mol/L PBS with 0.05% Tween-20) to remove unbound aptamer. The microplate wells were then blocked with blocking buffer (0.01 mol/L PBS with 3% BSA) for 1 h at 37 °C to prevent the appearance of nonspecific adsorption.

Then, a series of different concentrations of *B. bifidum* cells were added into each well for incubation at 37 °C for 45 min and the biotinylated aptamer and streptavidin-HRP were mixed at 37 °C for 30 min at the same time. After the microtiter plates were washed three times with the washing buffer, and 100 μL samples of the above biotinylated aptamer and streptavidin-HRP complexes were added to each well for reaction for 45 min at 37 °C. After washing, 200 μL TMB–H_2_O_2_ (tetramethyl benzidine-hydrogen peroxide) working solutions were added into each well for reaction without direct light exposure. After incubating for 15 min, the reaction was terminated with 50 μL 2 M H_2_SO_4_, and the OD at 450 nm was measured with the microplate reader.

## 4. Conclusions

This study is the first report of the use of whole-bacterium SELEX to identify ssDNA aptamers that are specific for *B. bifidum*. The results show that the aptamer CCFM641-5 bound tightly to *B. bifidum* with a *K*_d_ value in the nanomolar range, and could bind *B. bifidum* specifically over other bacterial species. According to the results of the present study, we demonstrate that the DNA aptamer CCFM641-5 can be used to capture and detect *B. bifidum*. Thus, the work described in this study testified the ability of this method to screen a good aptamer probe for the detection of *B. bifidum* and has the potential to contribute greatly to the development of the detection of *B. bifidum*.

In addition, the results from the experiments with aptamer truncations indicated that the 3′-primer and 5′-primer binding sites were important for an optimal binding affinity of the aptamer CCFM641-5 for *B. bifidum*. The experiments with proteinase treatment suggest that the component bound by the aptamer CCFM641-5 is likely protein on the *B. bifidum* cell surface.

Furthermore, we developed a colorimetric assay to detect *B. bifidum* which did not rely on expensive instrumentation, but on the basis of the aptamer CCFM641-5. The method is sensitive and specific and could be adopted to detect *B. bifidum* cells at concentrations as low as 10^4^ cfu/mL. Therefore, the colorimetric bioassay based on the aptamer CCFM641-5 is a promising method for the detection of *B. bifidum*.

## Figures and Tables

**Table 1:** Tested aptamer sequences ^a^.

Name	Sequence (5′ → 3′)
CCFM641-2	GCCTGGCCAGGTGCCCCGATATAGCGACGCCTTGCCCGGC
CCFM641-4	GCCCCGGACGGCGGGAAGCCTCGTACCCCCCGTGAGCGGC
CCFM641-5	TGCGTGAGCGGTAGCCCCGTACGACCCACTGTGGTTGGGC
CCFM641-12	GTCACACCGGCCGTCTCCGGTGTGGGACGCCCGCTGTGGC

^a^ The primer sequences are AGCAGCACAGAGGTCAGATG at the 5**′** end and CCTATGCGTGCTACCGTGAA at the 3**′** end.

**Table 2:** Full-length aptamer CCFM641-5 and truncated aptamer variants ^a^.

Name	Sequence (5′ → 3′)
CCFM641-5	AGCAGCACAGAGGTCAGATGTGCGTGAGCGGTAGCCCCGTACGACCCACTGTGGTTGGGCCCTATGCGTGCTACCGTGAA
CCFM641-5F	TGCGTGAGCGGTAGCCCCGTACGACCCACTGTGGTTGGGC CCTATGCGTGCTACCGTGAA
CCFM641-5R	AGCAGCACAGAGGTCAGATGTGCGTGAGCGGTAGCCCCGTACGACCCACTGTGGTTGGGC
CCFM641-5FR	TGCGTGAGCGGTAGCCCCGTACGACCCACTGTGGTTGGGC

^a^ The underlined sequences AGCAGCACAGAGGTCAGATG and CCTATGCGTGCTACCGTGAA are the primer binding sites.

## Supplementary Materials

Supplementary materials can be found at www.mdpi.com/1422-0067/18/5/883/s1.
