# Supplementary material for: Selection, Characterization and Interaction Studies of a DNA Aptamer for the Detection of Bifidobacterium bifidum
Source: Int J Mol Sci. 2017 Apr 25;18(5):883. doi: 10.3390/ijms18050883 (PMC5454810; doi:10.3390/ijms18050883)
Supplement: Supplementary file 1 [file ijms-18-00883-s001.pdf]

Supplementary Materials

Selection, Characterization and Interaction Studies of a DNA Aptamer for the Detection of *Bifidobacterium bifidum*

Lujun Hu, Linlin Wang, Wenwei Lu, Jianxin Zhao, Hao Zhang and Wei Chen

Table S1. Random sequence of seven families of 30 sequences [a].

Family	Name	Sequence (5′→3′)
Ⅰ	**CCFM641-2**	GCCTGGCCAGGTGCCCCGATATAGCGACGCCTTGCCCGGC
	**CCFM641-11**	GCCTGGCCAGGTGCCCCGATATAGCGACGCCTTGCCCGGC
	**CCFM641-23**	GCCTGGCCAGGTGCCCCGATATAGCGACGCCTTGCCCGGC
	**CCFM641-14**	GCCTGGCCAGGTGCGCCGATATAGCCTGCCCTTGCCCGGC
	**CCFM641-18**	GCCTGGCCACGTGCGCGGATATAGCCTGCCCTTGGCCGGC
Ⅱ	**CCFM641-4**	GCCCCGGACGGCGGGAAGCCTCGTACCCCCCGTGAGCGGC
	**CCFM641-9**	GCCCCGGACGGCGGGAAGCCTCGTACCCCCCGTGAGCGGC
	**CCFM641-25**	GCCCCGGACGGCGGGAAGCCTCGTACCCCCCGTGAGCGGC
	**CCFM641-28**	GCCCCGGACGGCGGGAAGCCTCGTACCCCCCGTGAGCGGC
	**CCFM641-7**	GCCCCGGACGGCGGGAAGCCTCGTACCCCGGGTGAGCGGC
Ⅲ	**CCFM641-5**	TGCGTGAGCGGTAGCCCCGTACGACCCACTGTGGTTGGGC
	**CCFM641-6**	TGCGTGAGCGGTAGCCCCGTACGACCCACTGTGGTTGGGC
	**CCFM641-17**	TGCGTGAGCGGTAGCCCCGTACGACCCACTGTGGTTGGGC
	**CCFM641-21**	TGCGTGAGCGGTAGCCCCGTACGACCCACTGTGGTTGGGC
	**CCFM641-26**	TGCGTGAGCGGTAGCCCCGTACGACCCACTGTGGTTGGGC
	**CCFM641-27**	TGCGTGAGCGGTAGCCCCGTACGACCCACTGTGGTTGGGC
	**CCFM641-30**	TGCGTGAGCGGTAGCCCCGTACGACCCACTGTGGTTGGGC
	**CCFM641-29**	TGCGTGAGCGGTAGCCCGGTACGACCCACTGTGGTTGGGC
	**CCFM641-3**	TGCGTGAGCGGTAGCCGGGTACGACCCACTGTGGTTGGGC
Ⅳ	**CCFM641-12**	GTCACACCGGCCGTCTCCGGTGTGGGACGCCCGCTGTGGC
	**CCFM641-13**	GTCACACCGGCCGTCTCCGGTGTGGGACGCCCGCTGTGGC
	**CCFM641-16**	GTCACACCGGCCGTCTCCGGTGTGGGACGCCCGCTGTGGC
	**CCFM641-22**	GTCACACCGGCCGTCTCCGGTGTGGGACGCCCGCTGTGGC
	**CCFM641-24**	GTCACACCGGCCGTCTCCGGTGTGGGACGCCCGCTGTGGC
	**CCFM641-19**	GTCACACGGCCCGTCTGCGGTGTGGGACGCCCGCTGTGGC
	**CCFM641-20**	GTCACACCGGCCGTCTCCGGTCTGGCACGCCGGCTGTGGC
Ⅴ	**CCFM641-8**	GCCCGCGCCCCTTCTGCCGTTGGTCGGAACCCCTGTGCGC
	**CCFM641-10**	GCCCGCGCGGGTTCTGCCGTTGGTGGGAACCCCTGTGCGC
Ⅵ	**CCFM641-1**	CGCCCTACACCACTCTCGGAGCGCTGTACGGCATCCCTGG
Ⅶ	**CCFM641-15**	GCCCGCACCACACACAGATGTCCATGTGTGCGCTGCCGGC

[a] The primer sequences are AGCAGCACAGAGGTCAGATG at the 5′ end and CCTATGCGTGCTACCGTGAA at the 3′ end.

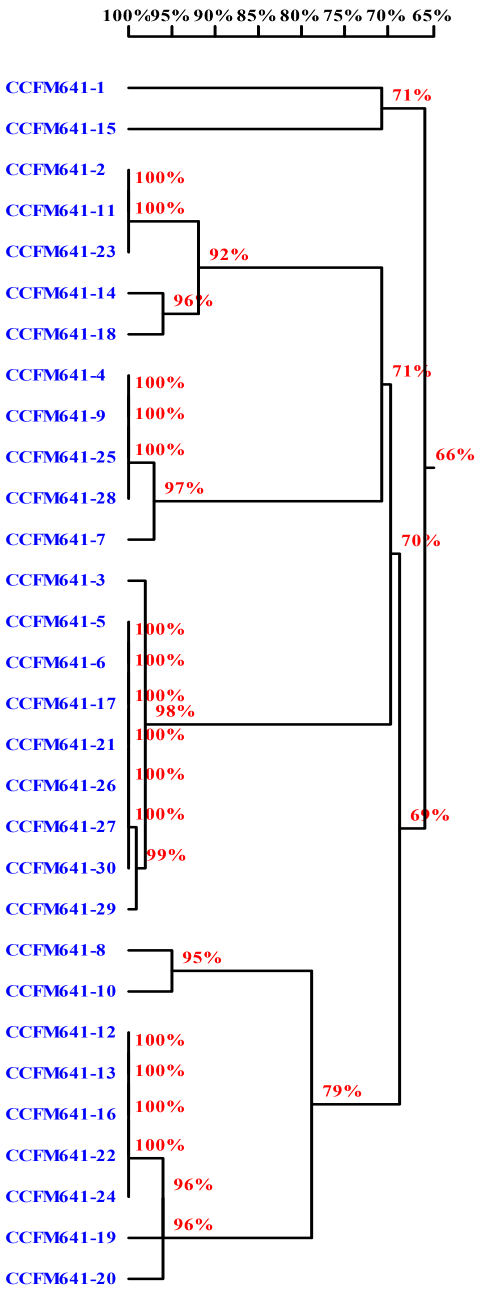

Figure S1. Homology tree of aptamer sequences. The sequence alignment was performed using the DNAMAN software package (Lynnon Biosoft Company, USA).

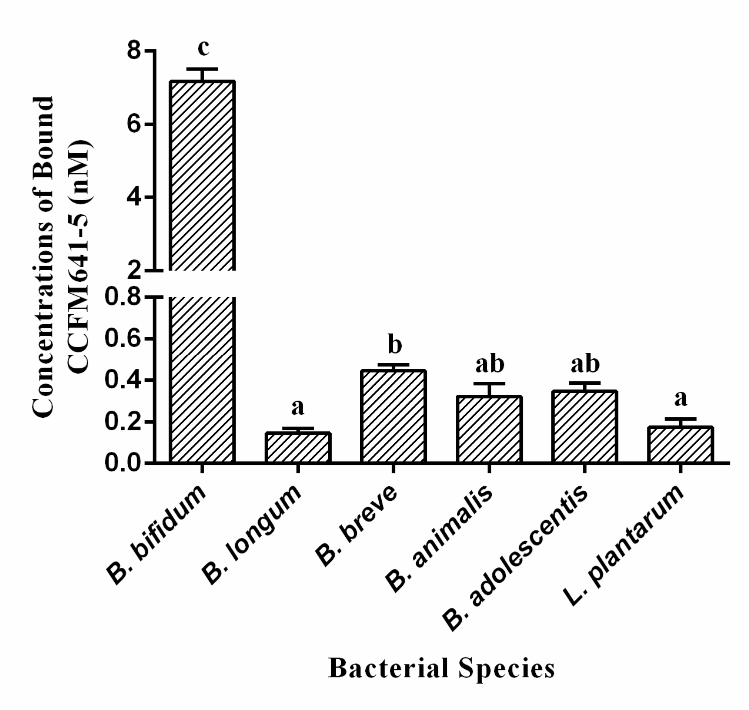

Figure S2. The binding abilities of aptamer CCFM641-5 to different bacterial species. Bound aptamer concentrations were quantified by qPCR. The concentrations of bound aptamer CCFM641-5 represent the mean ± SD of three independent experiments. Bars with different letters are significantly different ($p < 0.05$).
